# Ruminal background of predisposed milk urea (MU) concentration in Holsteins

**DOI:** 10.3389/fmicb.2022.939711

**Published:** 2022-09-13

**Authors:** Hanne Honerlagen, Henry Reyer, Dierck Segelke, Carolin Beatrix Maria Müller, Marie Christin Prahl, Siriluck Ponsuksili, Nares Trakooljul, Norbert Reinsch, Björn Kuhla, Klaus Wimmers

**Affiliations:** ^1^Research Institute for Farm Animal Biology (FBN), Institute of Genome Biology, Dummerstorf, Germany; ^2^IT-Solutions for Animal Production, Vereinigte Informationssysteme Tierhaltung w.V. (vit), Verden, Germany; ^3^Research Institute for Farm Animal Biology (FBN), Institute of Nutritional Physiology “Oskar Kellner”, Dummerstorf, Germany; ^4^Research Institute for Farm Animal Biology (FBN), Institute of Genetics and Biometry, Dummerstorf, Germany; ^5^Faculty of Agricultural and Environmental Sciences, University of Rostock, Rostock, Germany

**Keywords:** rumen microbiome, host gene expression, microbe–host relationship, dairy cow, milk urea

## Abstract

Efforts to reduce nitrogen (N) emissions are currently based on the optimization of dietary- N supply at average herd N requirements. The implementation of the considerable individual differences and predispositions in N- use efficiency and N- excretion in breeding programs is hampered by the difficulty of data collection. Cow individual milk urea (MU) concentration has been proposed as an easy-to-measure surrogate trait, but recent studies questioned its predictive power. Therefore, a deeper understanding of the biological mechanisms underlying predisposed higher (HMUg) or lower (LMUg) MU concentration in dairy cows is needed. Considering the complex N- metabolism in ruminants, the distinction between HMUg and LMUg could be based on differences in (i) the rumen microbial community, (ii) the host-specific transcription processes in the rumen villi, and (iii) the host–microbe interaction in the rumen. Therefore, rumen fluid and rumen epithelial samples from 10 HMUg and 10 LMUg cows were analyzed by 16S sequencing and HiSeq sequencing. In addition, the effect of dietary-N reduction on ruminal shifts was investigated in a second step. In total, 10 differentially abundant genera (DAG) were identified between HMUg and LMUg cows, elucidating greater abundances of ureolytic *Succinivibrionaceae_UCG-002* and *Ruminococcaceae*_*unclassified* in LMUg animals and enhanced occurrences of *Butyvibrio* in HMUg cows. Differential expression analysis revealed genes of the bovine Major Histocompatibility Complex (*BOLA* genes) as well as *MX1, ISG15*, and *PRSS2* displaying candidates of MU predisposition that further attributed to enhanced immune system activities in LMUg cows. A number of significant correlations between microbial genera and host transcript abundances were uncovered, including strikingly positive correlations of *BOLA-DRA* transcripts with *Roseburia* and *Lachnospiracea*e family abundances that might constitute particularly prominent microbial–host interplays of MU predisposition. The reduction of feed-N was followed by 18 DAG in HMUg and 19 DAG in LMUg, depicting pronounced interest on *Shuttleworthia*, which displayed controversial adaption in HMUg and LMUg cows. Lowering feed-N further elicited massive downregulation of immune response and energy metabolism pathways in LMUg. Considering breeding selection strategies, this study attributed information content to MU about predisposed ruminal N-utilization in Holstein–Friesians.

## Introduction

Nitrogen (N) excretions of dairy cows contribute substantially to N-deposition in the environment (Castillo et al., 2000). Considering the relevance for climate change (nitrous oxide (N_2_O) atmospheric emissions) and groundwater quality (nitrate (${\text{NO}}_{3}^{-}$) contamination), the dairy sector is demanded to reduce its N-emissions in the near future (Uwizeye et al., 2020). The primary source of N-emissions from dairy cows is represented by unutilized N, which goes into the cow *via* feed crude protein (CP) and is excreted *via* urine, milk and feces (Bryant, 1970; Abdoun et al., 2006). In this context, the ruminal conversion rate of feed N into valuable microbial protein, which is the result of rumen microbial growth, mainly determines the number of N-emissions (Tan et al., 2021).

In detail, the cow acquires N *via* the dietary CP. A small part of CP passes through the rumen as undegradable protein (UDP) and is absorbed in the small intestine or excreted *via* feces. The majority of dietary N from CP is provided to the rumen microbes for microbial growth. However, the rate of microbial N-utilization depends on various factors such as ruminal pH, dietary energy content, the absorption capacity of the rumen epithelium and the microbial community. Thus, a part of the ruminal N-influx is incorporated into microbial protein, whereas the remaining part is microbially converted to non-protein-N (NPN) in forms of ammonia (NH_3_), ammonium-ions (${\text{NH}}_{4}^{+}$), peptides and amino acids (Bryant, 1970; Hartinger et al., 2018; Cholewińska et al., 2020). The cow absorbs amino acids and peptides deriving from the microbial protein in the small intestine whereas the NPN-compounds are absorbed by the rumen epithelium. The amino acids and peptides can be utilized by the cow as valuable N-sources, while absorbed NH_3_ and ${\text{NH}}_{4}^{+}$ is transported with the blood stream to the liver. There, the ammonia is detoxified, yielding urea in an energy-intensive process. Urea is secreted back into the blood cycle from which it is eliminated *via* urine (urinary urea, UU) and milk (milk urea, MU), or secreted into the rumen either across the rumen epithelium or *via* saliva (Burgos et al., 2007; Spek et al., 2012). The recycled ruminal urea is converted by epithelium-bound, ureolytic rumen bacteria to CO_2_ and NH_3_. The latter, in turn, can be utilized by NH_3_-assimilating microbes for the metabolism of microbial protein (Jin et al., 2018).

In symbiosis with their microbes, dairy cows may utilize on average 25% of the dietary N (Calsamiglia et al., 2010). However, substantial variation in the N-utilization efficiency, which is defined as the ratio of gram N in product to gram N intake between individual cows has been observed, ranging from 15 to 40% (Calsamiglia et al., 2010; Spek et al., 2012; Gardiner et al., 2016; Chen et al., 2021). In recent years various studies have focused on the reduction of dietary CP levels to minimize N-emissions at herd level, but neither the cow-specific potential has been exploited intensely nor has the predisposition for individual N-use efficiency been considered for breeding progress yet (Cantalapiedra-Hijar et al., 2020; Bergen, 2021; Jahnel et al., 2021; Te Pas et al., 2021). One reason is that data collection for the cow-individual N-use efficiency is not feasible in huge cow cohorts. Generally, neither the cow individual N-uptake nor the content of N in urine and feces is measurable in practice operating dairy farms. Thus, in the last years the cow-specific MU concentration has been considered as a substitute trait (Burgos et al., 2007, 2010; König et al., 2008; Honerlagen et al., 2021; Jahnel et al., 2021). MU values are available to breeding companies at a large scale due to the results of monthly milk records. Furthermore, MU is moderately heritable and only weak or even no genetic correlations to further milk performance parameters (i.e., milk protein yield, milk fat yield) were evidenced (Wood et al., 2003; Miglior et al., 2007), which would, in principle, support MU as a trait for breeding selection. Due to the phenotypic correlation between MU, UU and blood urea concentration (Spek et al., 2012; Guliński et al., 2016) it has been hypothesized that breeding selection on lower MU concentration could lead to lower N-urine concentration and thus to lower N-emissions from dairy farming (Beatson et al., 2019). In addition, lower MU concentrations were also suggested as biomarkers for increased N-utilization efficiency (Nousiainen et al., 2004; Lavery and Ferris, 2021), being predominantly explained by enhanced ruminal N-utilization (Guliński et al., 2016). However, up to now the validity of MU as a substitute trait for cow-individual N-use efficiency and N-excretion is relying on the phenotypic correlation between MU and UU, but its utility has been questioned by the results of two recent studies (Correa-Luna et al., 2021; Müller et al., 2021). Thus, it became evident, that a deeper knowledge of the biological mechanisms underlying predisposed MU concentrations is indispensable before MU can be seriously proposed for breeding selection strategies.

Considering the large impact of ruminal processes on the N-metabolism in dairy cows, this study aims to elucidate ruminal mechanisms that distinguish between cows with predisposed higher (HMUg) and lower (LMUg) MU concentration to contribute to the validation of MU as substitutive trait for N-utilization efficiency and N-excretion. In detail, this study focuses on differences in (i) the rumen microbial community, (ii) the host-specific transcriptional processes in the rumen villi and (iii) the host–microbe interaction in the rumen between HMUg and LMUg cows.

Moreover, assumed that LMUg cows may possess lower N-input requirements as a consequence of more efficient N-utilization, which would result in an N-oversupply if fed a ration according to the recommendations, this study further investigated the effect of reduced dietary N-supply on the microbial community and on host specific rumen tissue processes in HMUg and LMUg cows.

## Materials and methods

Animal husbandry and sampling were carried out according to the guidelines of the German Animal Protection Law. All protocols were approved by the Institute's Animal Welfare Commission. The experimental protocol is in strict compliance with the German Animal Welfare Legislation, and has been approved by the Ethics Committee of the Federal State of Mecklenburg, Western Pomerania, Germany (Landesamt für Landwirtschaft, Lebensmittelsicherheit und Fischerei; LALLF M-V7221.3-2-019/19) and is in accordance with the ARRIVE guidelines.

### Cow population and sample collection

The predisposition of cows for a higher or lower MU concentration (HMUg and LMUg) was determined by deregressed proofs of an estimated breeding value for MU (EBVMU). The EBVMUs were estimated by vit Verden (Vereinigte Informationssysteme Tierhaltung, Verden, Germany) applying the established model for somatic cell score in milk [Vereinigte Informationssysteme Tierhaltung (VIT)., (2020, access 20.01.2020)]. The estimation was based on a milk record dataset of around 8 million lactation curves of milk urea values from Holstein cows in their first to third parities. The deregression of EBVMUs is a pre step for genomic breeding value estimation. The HMUg was determined as predisposition by positive values, LMUg by negative values. Based on this categorization, 10 HMUg and 10 LMUg cows were housed in pairs, comprising one HMUg and one LMUg cow, respectiveley. The phenotypes of MU concentration were obtained from the recent five milk records depicting HMUg = 278.1 ± 15.8 (mean ± SD) mg/L and LMUg = 181.54 ± 15.08 (mean ± SD) mg/L with comparable milk yields between the groups.

These cow groups were fed an isocaloric diet [10.1 ± 0.2 MJ metabolisable energy/kg dry matter (DM)], with either recommended (normal) CP content (NP with 157 ± 2.68 (mean ± SD) g CP/kg DM) or low CP content [LP with 139 ± 8.37 (mean ± SD) g CP/kg DM], resulting in a 4 ×5 balanced design (NPxHMUg, NPxLMUg, LPxHMUg, LPxLMUg; each *n* = 5). The experiment was conducted in 10 trials, with each trial comprising one cow pair (HMUg and LMUg), which was fed either the NP or the LP diet. The LP and NP rations were offered twice-daily for *ad libitum* intake as a total mixed ration. A detailed description of the diet, the feeding scheme as well as phenotypic analyses of N-metabolites (i.e., ammonia concentration in the rumen fluids, plasma urea, and glutamine concentration) is documented in Müller et al. (2021). After 2 weeks, the cows were slaughtered 4 h after morning milking and feeding by exsanguination after captive bolt stunning. Samples of rumen villi from the *saccus dorsalis* containing all rumen villus cell layers were collected, i.e., the basal membrane cells, which connect villi to blood stream and muscle tissue. The samples were rinsed, snap frozen in liquid nitrogen and stored at −80°C until RNA isolation. In addition, the rumen fluids (50 ml) were collected as a homogeneous mixture of the entire rumen content and stored at −20° until isolation of microbial DNA.

### Rumen fluids: Microbial DNA extraction, 16S rRNA amplicon sequencing, and data preparation

The rumen fluid samples (50 ml) were mixed and microbial DNA was extracted with the PowerLyzer PowerSoil DNA isolation kit (QIAGEN, Hilden, Germany) following manufacture's recommendations, but utilizing 800 μl rumen fluid sample (instead of 250 μl) and two additional heat incubation steps (step 1: 70°C, 10 min; step 2: 95°C, 10 min) before the bead beating procedure. The quantity of the extracted DNA was determined on NanoDrop ND-2000 (Thermo Fisher Scientific, Dreieich, Germany) and afterwards diluted to 10 ng/μl per sample. The diluted DNA extracts were utilized to amplify the V4 hyper-variable region of the 16S rRNA gene. The specific primer enclosed the V4-specific sequence, the sequencing flow cell adapter and a specific barcode for each primer as previously described (Kozich et al., 2013). The amplification was conducted in duplicate by polymerase chain reaction (PCR) with initial denaturation at 95°C for 2 min, followed by 35 cycles at 95°C for 30 s, 50°C for 60 s, and 72°C for 90 s, and a final extension for 10 min at 72°C using the 5 Prime HotMasterMix (5 Prime, Hamburg, Germany). The PCR products (16S amplicons) were checked on agarose gel and subsequently purified, normalized and pooled for sequencing using the SequalPrep normalization plate kit (Thermo Fisher Scientific, Dreieich, Germany) The sequencing of the amplicons was conducted on HiSeq2500 (Illumina, San Diego, CA) with 250 bp paired-end reads.

The sequence data were then trimmed for the adapter sequences and filtered with mothur software for sequences that exceeded 275 bp in length, which contained ambiguous base calls or excessively long homopolymers (>8 bases), as well as putative chimeras were removed [version 1.44.1 (Schloss et al., 2009)].The sequences were further globally aligned to the Silva reference database (release 138; https://www.arb-silva.de/, access 10.6.20) and subsequently aggregated into operational taxonomic units (OTU) considering a sequence identity of ≥97%. Taxonomic annotations for OTUs were obtained from the Silva database (release 138). Considering stratification by dispersion, the count data was rarefied to the sample with the lowest read depth (745,235 reads) as suggested by Mcmurdie and Holmes (2014).

### Rumen fluids: Statistical analysis of 16S rRNA sequencing data

Initially, very low abundant OTUs with <10 counts in all 20 cows were pre-discarded from further analyses. The microbial community in rumen fluids was analyzed with inverse Simpson diversity index (alpha diversity) at OTU level utilizing the R packages “vegan” (Oksanen et al., 2013) and “agricolae” (De Mendiburu Delgado, 2009). Means of Simpson indices of the predisposition groups (HMUg and LMUg) and the diet groups (NP and LP in HMUg and LMUg cows, respectively) were tested for significant differences by applying Student's *t*-test. The subsequent analyses were conducted at genus level, excluding genera with <10 counts in 5 or more cows to avoid potential noise by barely observed reads. The remaining dataset was tested for significantly differentially abundant genera (DAG) between the groups (HMUg–LMUg; NPxHMUg–LPxHMUg; NPxLMUg–LPxLMUg) utilizing Wald test in the DESeq2 R package (Love et al., 2014). For the contrast of predisposition (HMUg–LMUg) the trial was implemented as further fixed effect in the statistical model, accounting for different CP levels in the feed ration. For the differences between the diets, the contrasts within each predisposition group were analyzed and expressed as fold changes obtained from DESeq2 delogarithmized log2fold changes. Genera with *p*-values below 0.05 were considered as DAG.

### Rumen tissue: RNA extraction, library preparation, sequencing, and data preparation

The total RNA from homogenized rumen villi was isolated using TRI reagent (Sigma–Aldrich, Taufkirchen, Germany), followed by DNaseI treatment (Roche, Mannheim, Germany) and a subsequent column-based purification step utilizing the NucleoSpin RNA Kit (Macherey-Nagel, Düren, Germany). The RNA quantity was measured on NanoDrop ND-2000 (Thermo Fisher Scientific) and further assessed for integrity by application on agarose gel and *via* 2100 Bioanalyzer system (Agilent, Santa Clara, CA). The RNA library preparation was performed using the TruSeq Stranded mRNA kit according to the manufacture's recommendation (Illumina). The resulting libraries were quality checked on the 2100 Bioanalyzer system and quantified using the Invitrogen Qubit dsDNA HS kit (Thermo Fisher Scientific). Libraries were paired-end sequenced for 2 ×71 cycles on HiSeq 2500 (Illumina). The raw sequencing reads (fastq) were quality-checked utilizing FastQC, version 0.11.8 (http://www.bioinformatics.babraham.ac.uk/projects/fastqc/). The data preprocessing was conducted with Trim Galore v.0.6.5 (https://www.bioinformatics.babraham.ac.uk/projects/trim_galore/). The low-quality reads (mean Q-score <20) and short reads (<20 bp) were filtered out. Adapter-like sequences at the 3'-end of sequence reads were trimmed. The remaining high quality paired-end reads comprised a read depth of 20,324,785 ± 3,234,163 (mean ± SD) per sample and were further aligned to the current reference genome, Bos_taurus.ARS-UCD1.2 (access 01. 08.2020) with an average alignment rate of 97.86% ± 0.01% (SD) using Hisat2, version 2.2.0 (Kim et al., 2015, 2019; Pertea et al., 2016). The number of reads uniquely mapped to each gene were extracted from the HISAT2 mapping results using HTSeq, version 0.12.4 (Anders et al., 2015).

### Rumen tissue: Statistical analysis of RNAseq data

The RNA dataset was analyzed for significantly differentially expressed genes (DEG) between groups—corresponding to the 16S microbiota sequence data (HMUg–LMUg; NPxHMUg–LPxHMUg; and NPxLMUg–LPxLMUg)—by utilizing Wald test in DESeq2 package (Love et al., 2014). Genes with <10 counts in 10 or more cows were excluded from analysis, leading to a dataset of 12,627 genes. The trial was considered as further fixed effect for HMUg–LMUg contrast. Genes with a Benjamini–Hochberg adjusted *p*-values <0.05 were considered as DEG. The DEG identifiers were extracted to investigate the biological function of the respective gene using the databases GeneCards (http://www.genecards.org), Ensembl (http://www.ensembl.org), and the published literature. Furthermore, genes revealing *p*-values below 0.01 (corresponding adjusted *p*-values <0.6) were imported into Ingenuity Pathway Analysis (IPA; Ingenuity® Systems, http://www.ingenuity.com) for enrichment analysis between the groups (HMUg–LMUg; NPxHMUg–LPxHMUg; NPxLMUg–LPxLMUg). Pathways with an adjusted *p*-value <0.05 were defined as significantly enriched between the groups. Significant pathway inhibition or activation was defined by *Z*-scores (significant inhibition: *Z*-score < -2; significant activation: *Z*-score >2) in IPA. The *Z*-scores between −2 and 0 or 0 and 2 were considered indicators of inhibition or activation, respectively. The pathways were further assigned to general functions after evaluation in Targeted Explorer (IPA). The pathways of cancer and disease were excluded.

### Integration of microbial and transcriptome data: Statistical analysis

Datasets of rumen microbial genera (*n* = 229 microbes) and gene expression (*n* = 12,627 genes) were initially transformed by variance-stabilizing transformation (DESeq2 R package). Subsequently, sparse Partial Least Squares Discriminant Analysis (sPLS-DA) was applied on both datasets. The sPLS-DA is incorporated in R package “mixOmics” (version 6.6.2, available at: http://mixomics.org) (Rohart et al., 2017) and achieves feature selection and dimension reduction simultaneously in dependence on a specific trait (Chun and Keleş, 2010). In this case, sPLS-DA was utilized to select a key subset of microbial genera and a key subset of genes that discriminate the microbial community and the rumen tissue transcriptome between HMUg and LMUg cows on two components each. The discrimination by the generated subsets was visualized in two plots.

The data subsets were further analyzed for microbial abundance–gene transcript correlations by utilizing Pearson statistics. Microbe–gene pairs exhibiting *p*-values below 0.01 were considered as significantly correlated. The results were visualized in a correlation map using R package “pheatmap” (version 1.0.12, available at: https://cran.r-project.org/web/packages/pheatmap) (Kolde and Kolde, 2015). The correlation map identified two dominant clusters (I and II). The genes attributing to the respective clusters were subsequently analyzed for significantly enriched pathways between HMUg and LMUg predisposition in IPA (adjusted *p* <0.05).

## Results

The microbial community in rumen fluids comprised 7,697 OTUs after filtering, which attributed to 24 phyla, 45 orders, 101 classes, 168 families, and 350 genera across all 20 cows. The filtered transcriptome dataset constituted 12,767 genes.

### Microbial analyses

**Figure 1:**
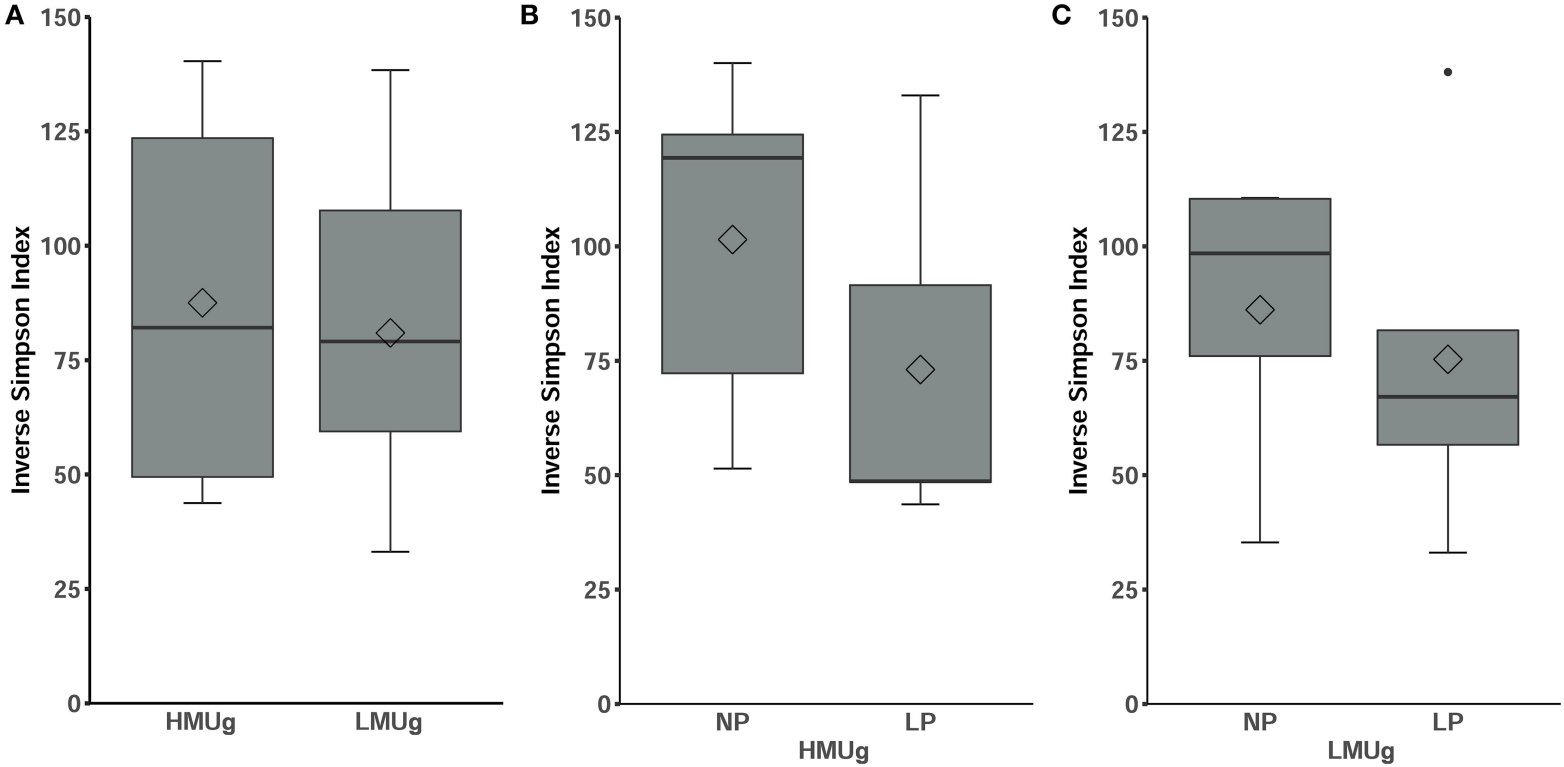
Alpha diversity of the microbial community in rumen fluids of **(A)** HMUg and LMUg cows and after lowering dietary N in HMUg **(B)** and LMUg **(C)** cows, respectively. Groups are displayed on the *x*-axis; inverse Simpson values scale the *y*-axis.

The microbial composition ranked at inverse Simpson values of 87.51 ± 39.13 (mean ± SD) in HMUg cows and 80.95 ± 34.24 (mean ± SD) in LMUg cows, indicating no significant differences between the predispositions (Figure 1A). With lower dietary CP level, alpha diversity numerically decreased, but not significantly, neither in HMUg nor in LMUg cows (Figures 1B,C).

The DESeq2 analysis at genus level revealed 10 DAG between HMUg and LMUg cows, which covered relative abundances between 0.0016 and 2.3668% across all cows (Table 1). Notably, the six most abundant genera (*Prevotella, Rikenellaceae_RC9_gut_group, Methanobrevibacter, Christensenellaceae_R-7_group, F082_ge*, and *NK4A214_group*) captured together 51.6% of total rumen abundances and did not differ significantly between HMUg and LMUg cows. Within the DAG, *Unclassified Ruminococcae* exhibited the broadest relative abundance (2.3668%) and manifested a 1.39-fold higher presence in LMUg compared to HMUg animals, whereas *Butyrivibrio* was significantly more abundant in HMUg compared to LMUg cows. Moreover, *Lachnospiraceae_UCG-010* showed significantly higher occurrence in HMUg compared to LMUg, while *Succinivibrionaceae_UCG-002* displayed a 2.24-fold higher occurrence in LMUg than in HMUg animals. However, highest fold changes were observed for *Acetobacter* and *Monoglobus*, with *Acetobacter* depicting 3.25-fold more abundance in HMUg and *Monoglobus* being 2.33-times more prevalent in LMUg animals.

**Table 1:** Significantly differentially abundant genera (DAG) in the rumen of HMUg and LMUg cows.

	**Relative abundance**^**a**^ **(%)**			
**Genus**	**HMUg**	**LMUg**	**Fold change^**b**^**	***p*-value^**c**^**
Acetobacter*	0.0021	0.0010	3.25	0.0450
*Acholeplasmataceae_unclassified*	0.0036	0.0075	−2.02	0.0163
*Butyrivibrio*	0.3949	0.2594	1.37	0.0206
*CAG-352*	0.3157	0.4505	−1.95	0.0272
*Catenibacterium*	0.0015	0.0026	−1.71	0.0353
Lachnospiraceae_UCG-010*	0.0105	0.0060	1.87	0.0418
*Monoglobus*	0.0121	0.0207	−2.33	0.0021
*Ruminococcaceae_unclassified*	1.9833	2.7502	−1.39	0.0440
*Subdoligranulum*	0.0139	0.0195	−1.42	0.0471
*Succinivibrionaceae_UCG-002*	0.4456	0.8744	−2.24	0.0336

^*a*^Calculated as mean of the respective group.

^*b*^Fold changes derive from DESeq2 analysis including trial as fixed effect in the model and refer to HMUg compared to LMUg.

^*c*^*p*-values <0.05 indicating significance.

*Displays genera that were also identified as DAG in comparison between NP and LP diets within HMUg and LMUg groups (Table 2).

For the comparison of NP and LP diets, 18 DAG were identified in HMUg and 19 DAG in LMUg cows, including four DAG that were previously identified in HMUg–LMUg comparison (Table 2). The reduction of dietary CP content was generally accompanied by high fold changes of genera abundances. For instance, *MVP-15_ge* abundances decreased 133-fold in HMUg, whereas *Sharpea* abundances increased 24-fold in HMUg and 166-fold in LMUg with LP feeding. *Incertae sedis* was less abundant in both, HMUg and LMUg animals, when cows were exposed to the LP diet. Furthermore, the abundances of *Shuttleworthia* increased in HMUg, but decreased in LMUg cows with the LP diet. Following dietary CP reduction, HMUg animals exhibited a further increase of *Acidaminococcus* abundances and of three genera belonging to phylum *Desulfobacterota* (*Desulfovibrio, Desulfobulbus, Desulfobulbaceae_unclassified*) with mean relative abundances between 0.0015 and 0.0993%. In LMUg animals, the significant increases of *Streptococcus* and *Lachnospiraceae_NK3A20_group* abundances were observed on the LP compared to NP ration. On the contrary, *Alphaproteobacteria_unclassified* and *Succinivibrionaceae_unclassified* abundances decreased remarkably under conditions of LP feeding in LMUg cows at 4.05- and 13.3-times, respectively. Interestingly, *Acetobacter*, whose abundance was significantly lower in LMUg than in HMUg cows (Table 1), showed a further 21-fold decrease in LMUg cows upon the reduction of the dietary CP content (Table 2).

**Table 2:** Significantly differentially abundant genera (DAG) in the rumen comparing normal crude protein (NP) and low crude protein (LP) diet offered to HMUg and LMUg cows.

	**Relative abundance**^**a**^ **(%)**	
**Genus**	**NP**	**LP**	**Fold change^**b**^**	***p*-value^**c**^**
HMUg
*Acidaminococcus*	0.0026	0.0282	−11.52	0.0008
*Bacilli_unclassified*	0.1169	0.0568	1.80	0.0123
*Cardiobacteriaceae_unclassified*	0.0217	0.0586	−3.08	0.0361
*Desulfobulbaceae_unclassified*	0.0007	0.0029	−4.43	0.0056
*Desulfobulbus*	0.0445	0.1101	−2.71	0.0128
*Desulfovibrio*	0.0742	0.1605	−2.55	0.0087
*horsej-a03*	0.0148	0.0015	9.57	0.0395
*Incertae_Sedis*	0.0144	0.0017	6.54	0.0005
Lachnospira*	0.0076	0.0013	4.77	0.0049
*Lachnospiraceae_UCG-009*	0.0049	0.0008	5.31	0.0051
*M2PT2-76_termite_group*	0.0339	0.0035	7.57	0.0316
*MVP-15_ge*	0.0136	0.0001	132.57	0.0001
*Prevotellaceae_Ga6A1_group*	0.0757	0.0118	5.36	0.0073
*Pseudobutyrivibrio*	0.2100	0.0293	6.22	0.0368
Sharpea*	0.0015	0.0369	−23.72	0.0055
Shuttleworthia*	0.0178	0.1586	−10.20	0.0001
*Syntrophococcus*	0.0855	0.1888	−2.38	0.0411
*vadinBE97_ge*	0.1724	0.0333	4.71	0.0288
LMUg
Acetobacter*	0.0019	0.0001	21.31	0.0068
*Alphaproteobacteria_unclassified*	0.0158	0.0037	4.05	0.0374
*Anaerolineae_unclassified*	0.0654	0.3116	−6.11	0.0028
*Anaerovibrio*	0.0153	0.0048	2.93	0.0085
*COB_P4-1_termite_group_ge*	0.0096	0.0004	18.24	0.0002
*Hungateiclostridiaceae_unclassified*	0.0092	0.0222	−2.64	0.0244
Incertae_Sedis*	0.0139	0.0016	7.35	0.0002
Lachnospira*	0.0079	0.0020	3.43	0.0250
*Lachnospiraceae_NK3A20_group*	1.1391	2.8116	−2.88	0.0264
Lachnospiraceae_UCG-010*	0.0100	0.0020	4.31	0.0078
*Pirellulaceae_unclassified*	0.0043	0.0131	−3.74	0.0048
*Ruminiclostridium*	0.0186	0.0476	−2.78	0.0102
Sharpea*	0.0001	0.0119	−166.22	0.0001
Shuttleworthia*	0.0600	0.0077	6.45	0.0023
*Slackia*	0.0009	0.0044	−5.46	0.0472
*Streptococcus*	0.0057	0.0206	−4.44	0.0071
*Succinivibrionaceae_unclassified*	0.0092	0.0006	13.29	0.0027
*UCG-007 (Oscillospiraceae)*	0.0075	0.0008	7.92	0.0154
*UCG-012 (Hungateiclostridiaceae)*	0.0027	0.0003	7.00	0.0213

^*a*^Calculated as mean of the respective group.

^*b*^Fold changes derived from DESeq2 analysis and refer to NP compared to LP.

^*c*^*p*-values <0.05 indicating significance.

*Displays genera that were identified significantly differential abundant in more than one contrast.

### Transcriptome analyses

In the filtered transcriptome dataset, 28 genes were identified as differentially expressed in the rumen between HMUg and LMUg cows (Supplementary material 1, p. 1). The highest fold changes were observed for Matrix Metallopeptidase 3 (*MMP3*) and Transition Protein 2 (*TNP2*), with higher transcript abundances in HMUg and LMUg, respectively. Furthermore, 166 genes passed the relaxed significance threshold (*p* <0.01) for enrichment analysis (Supplementary material 1, p. 2) and revealed 13 significantly enriched pathways that contributed almost exclusively (9 out of 13) to the immune system (Table 3). Antigen Presentation Pathway revealed the most prominent adjusted *p*-value. In addition, Interferon Signaling and Neuroinflammation Signaling pointed as two further candidates, displaying significant inhibition in HMUg. Whereas, Ubiquitin Like Modifier 15 (*ISG15*) and MX Dynamin Like GTPase 1 (*MX1*) were included in the former, *MMP3* and Prostaglandin-Endoperoxide Synthase 2 (*PTGS2*) contributed to the latter. Interestingly, these four genes also ranked under the top 25 genes in terms of absolute fold changes among all annotated genes in the filtered dataset (Supplementary material 1). However, Serine Protease 2 (*PRSS2*) and UL16 Binding Protein 17 (*ULBP17*) displayed the most prominent fold changes.

**Table 3:** Significantly enriched pathways in rumen tissue comparing HMUg and LMUg cows.

**Canonical pathways^a^**	**General function**	**-log(*p*-value)^b^**	**Genes**
Antigen presentation pathway	IS	7.11	*CD74,BOLA-B,BOLA-DMA,BOLA-DMB,BOLA-DOB,BOLA-DRA,PSMB8,TAPBP*
*Interferon signaling*	IS	6.19	*IRF1,IRF9,**ISG15**,**MX1**,**OAS1Y**,PSMB8,STAT2*
*Complement system*	IS	6.19	*C1QA,C1QB,C1R,C1S,C2,CFB,SERPING1*
IL-4 signaling	IS	4.91	*BOLA-B,BOLA-DMA,BOLA-DMB,BOLA-DOB,BOLA-DRA,IL2RG,**IRF4**,JAK3*
B cell development	IS	3.38	*BOLA-B,BOLA-DMA,BOLA-DMB,BOLA-DOB,BOLA-DRA*
*Neuroinflammation signaling pathway*	IS	2.69	*CXCL12,BOLA-B,BOLA-DMA,BOLA-DMB,BOLA-DOB,BOLA-DRA,IRF7,JAK3,**MMP3**,**PTGS2***
Acetate conversion to Acetyl-CoA	FM/EM	2.17	*ACSS1,ACSS2*
Primary immunodeficiency signaling	IS	1.91	*CD3E,CD4,IL2RG,JAK3*
Glucocorticoid receptor signaling	IS	1.76	*CD3E,BOLA-B,BOLA-DMA,BOLA-DMB,BOLA-DOB,BOLA-DRA,IL2RA,IL2RG,JAK3,**MMP3**,**PTGS2**,**TSC22D3***
*Activation of IRF by cytosolic pattern recognition receptors*	IS	1.67	*IRF7,IRF9,**ISG15**,STAT2*
Ethanol degradation II	FM/EM	1.63	*ACSS1,ACSS2,AKR1A1*
Glutathione-mediated detoxification	CP	1.62	*GSTA1,**GSTA2**,GSTA4*
Adipogenesis pathway	FM/EM	1.41	*BMP2,CEBPD,FABP4,WNT5A,XBP1*

^*a*^Terms obtained from Ingenuity Pathway Analysis.

^*b*^Benjamini–Hochberg adjusted *p*-values <0.05 indicating significance; underlined italic letters display significant inhibition in HMUg (z-score < −2), italic letters indicate predicted inhibition in HMUg (z-score < −2 to 0); bold letters emphasize genes that display fold changes ranking in the top 25 of the filtered transcriptome datasets.

IS, immune system; FM/EM, fat/energy metabolism; CP, cell protection.

Considering the reduction of dietary CP in HMUg cows, no DEGs were observed, but 68 genes passed the relaxed significance threshold for pathway analysis. This list culminated in the identification of Intrinsic Prothrombin Activation Pathway and MSP-RON Signaling Pathway as significantly enriched (Table 4; Supplementary material 2, p. 1). In contrast, LMUg animals showed 1,220 DEGs and 139 significantly enriched pathways when dietary CP content was reduced (Table 4; Supplementary material 2, p. 2, 3). Whereas, Oxidative Phosphorylation and Semaphorin Neuronal Repulsive Signaling Pathway were significantly activated when LMUg cows were fed the LP ration, additional 79 pathways were identified to be significantly inhibited, mainly representing pathways of immune response and energy metabolism as well as pathways that contribute to cell cycle and cell maintenance (Table 4).

**Table 4:** Significantly enriched pathways between normal protein (NP) and low protein (LP) diet in rumen tissue of HMUg and LMUg cows.

	**Canonical pathways^a^**	**General function**	***z*-score**	**-log(adj. *p*-value)^b^**
HMUg
	Intrinsic prothrombin activation pathway	IS		2.9
	MSP-RON signaling pathway	IS		2.75
LMUg
	*Insulin receptor signaling*	EM	−2.294	4.49
	*PI3K signaling in B lymphocytes*	IS	−3.128	4.16
	*CNTF signaling*	IS	−2.673	4.07
	*RAN signaling*	CC	−2.828	4.07
	Virus entry *via* endocytic pathways	IS		3.91
	Protein ubiquitination pathway	CC		3.82
	*GM-CSF signaling*	IS	−2.138	3.82
	*UVA-induced MAPK signaling*	CC	−2.887	3.82
	*p70S6K signaling*	CF	−2.982	3.64
	*Kinetochore metaphase signaling pathway*	CC	−1.698	3.47
	*Signaling by Rho family GTPases*	CM	−1.8	3.37
	*IL-3 signaling*	IS	−2.324	3.36
	*Aldosterone signaling in epithelial cells*	CS	−1.941	3.33
	Ephrin A signaling	CS		3.22
	*IGF-1 signaling*	EM	−2.138	3.18
	Semaphorin neuronal repulsive signaling pathway*	CM	2.065	2.54
	Oxidative phosphorylation*	EM	2.84	2.39

^*a*^Terms obtained from Ingenuity Pathway Analysis.

^*b*^Benjamini–Hochberg adjusted *p*-values <0.05 indicating significance; underlined italic letters indicate significant activation (z-score>2) or inhibition (z-score < −2) under NP, italic letters indicate predicted inhibition (z-score < −2 to 0) under NP; for LMUg only the top 15 (adjusted *p*-value) significantly inhibited pathways and the two significantly activated pathways (*indicates activation) under NP are displayed.

IS, immune system; FM/EM, fat/energy metabolism; CP, cell protection; CC, cell cycle; CF, cell function; CM, cell maintenance; CS, cell signaling.

### Integration of microbial and transcriptome data

**Figure 2:**
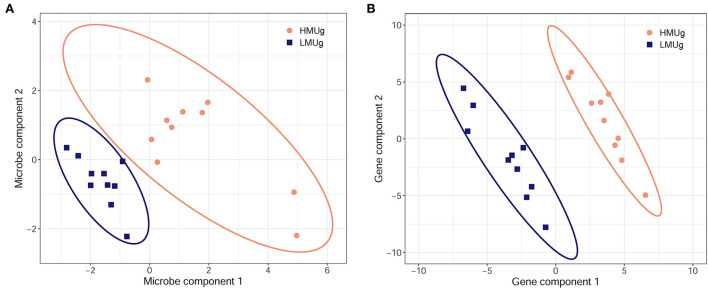
Sparse partial least square-discriminant analysis of HMUg and LMUg cows, driven by **(A)** 53 key microbial genera in the rumen fluid and by **(B)** 100 key genes in the rumen tissue.

The multivariate discriminant analysis (sPLS-DA) revealed a microbial subset comprising 50 genera in the first component and 5 genera in the second component separating HMUg and LMUg cows' rumen microbial communities (Figure 2A; Supplementary material 3, p. 1). Two genera contributed to both components, giving a subset of 53 genera that distinguished HMUg and LMUg cows. Eight genera of the subset were also identified as DAG in HMUg–LMUg contrast. The transcriptome subset included 100 genes, comprising 50 genes in each component (Figure 2B; Supplementary material 3, p. 2). Twelve genes thereof were uncovered as DEG between HMUg and LMUg.

**Figure 3:**
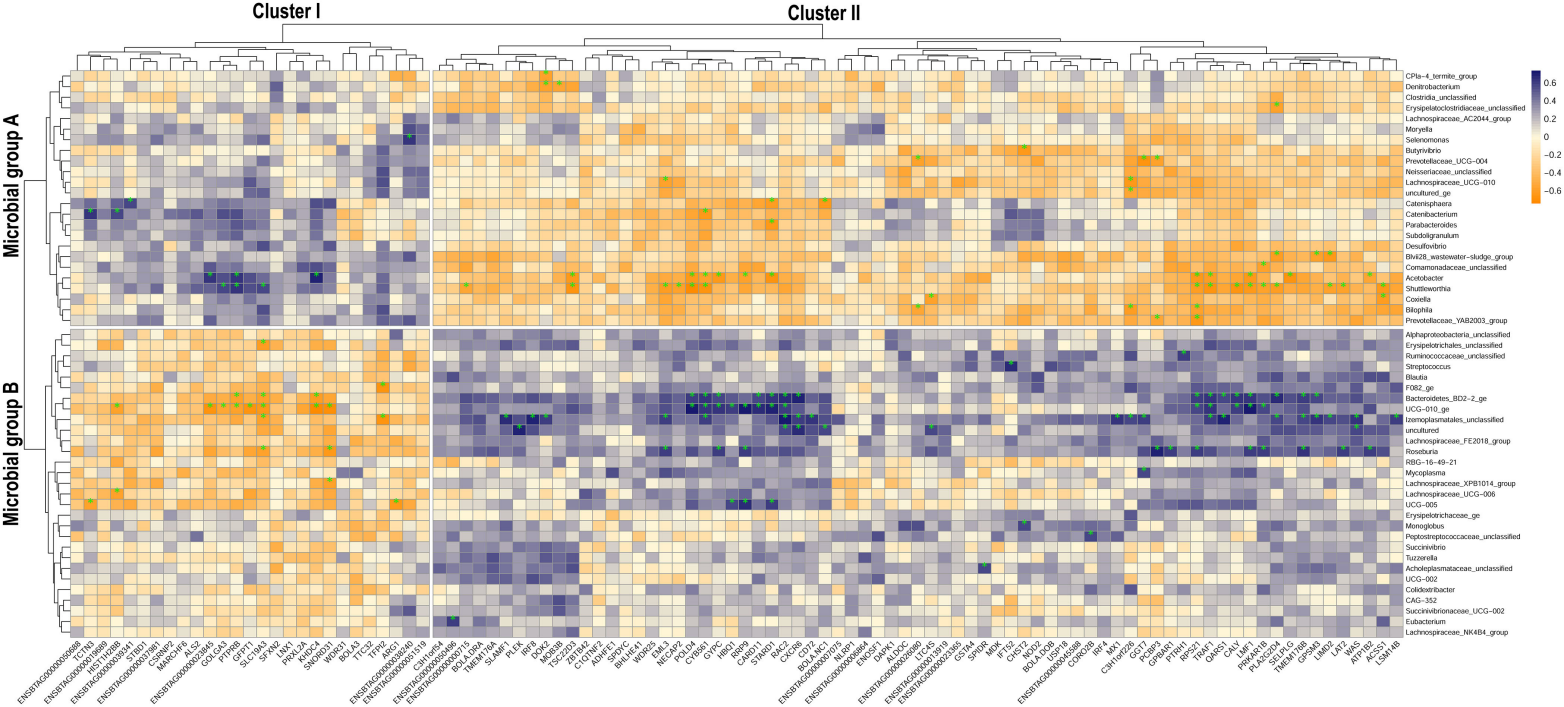
Correlation map of ruminal host-microbiota interaction depicting the relationship of microbial subset abundances and gene subset transcripts that separate HMUg and LMUg rumen profiles. Subsets derived from sPLS-DA analysis; estimated correlations are based on the microbial abundances and gene transcripts in HMUg and LMUg cows; *indicates significant correlation (*p* <0.01).

The correlation analysis between the microbial and the gene subset separating HMUg and LMUg cows detected 157 significantly correlated microbial genera–gene pairs. The two pronounced clusters I and II were generated by two groups of microbes (A and B) and two groups of genes (I and II) that correlated contrarily (Figure 3; Supplementary material 3). In detail, Cluster I depicted gene Group I (27 genes) being positively correlated to microbial Group A (24 genera) and negatively correlated to microbial Group B (29 genera). Cluster II on the other hand showed negative correlations between gene Group II (73 genes) and microbial Group A, but positive correlations between gene Group II and microbial Group B.

The subsequent pathway analysis of gene Groups I and II revealed significant enrichment of Urea Cycle, Arginine Degradation and Citrulline Metabolism for Group I, which was mainly determined by Arginase 1 (*ARG1*) expression (Table 5). The *ARG1* expression was negatively correlated with *UCG-005 abundances* (*r* = −0.64, *p* <0.01; Figure 3; Supplementary material 4). Furthermore, the differential expression of Glutamine-fructose-6-phosphate transaminase 1 (*GFPT1*) in Group I contributed to the significant enrichment of UDP-N-acetyl-D-glucosamine biosynthesis II (Table 5). Moreover, *GFPT1* was negatively correlated with *UCG-010_ge* (*r* = −0.68, *p* <0.01), *Lachnospiraceae_UCG-006* (*r* = −0.56, *p* = 0.011), and *Roseburia* abundances (*r* = −0.56, *p* = 0.01) (Figure 3; Supplementary material 4). A higher expression level of *GFTP1* was accompanied by increased abundances of *Lachnospiraceae_UCG-010* (*r* = 0.52, *p* = 0.02), *Acetobacter* (*r* = 0.56, *p* = 0.01), and *Shuttleworthia* (*r* = 0.55, *p* = 0.01), which had been pre-identified as DAG in the comparison of HMUg and LMUg cows (Figure 3; Table 1; Supplementary material 4).

**Table 5:** Significantly enriched pathways between HMUg and LMUg cows based on gene Groups I and II derived from ruminal host-microbiota interaction analysis (Figure 3).

**Ingenuity canonical pathways^a^**	**–log(*p*-value)^b^**	**Genes**	**Ratio^c^**	**Cluster**
Urea cycle	1.85	*ARG1*	0.17	Group I
Arginine degradation VI (Arginase 2 Pathway)	1.85	*ARG1*	0.17	Group I
UDP-N-acetyl-D-glucosamine biosynthesis II	1.85	*GFPT1*	0.17	Group I
Arginine degradation I (arginase pathway)	1.85	*ARG1*	0.25	Group I
Citrulline biosynthesis	1.78	*ARG1*	0.11	Group I
Superpathway of citrulline metabolism	1.63	*ARG1*	0.07	Group I
B cell development	1.87	*BOLA-B,BOLA-DOB,BOLA-DRA*	0.07	Group II
IL-4 signaling	1.87	*BOLA-B,BOLA-DOB,BOLA-DRA,IRF4*	0.043	Group II
Antigen presentation pathway	1.87	*BOLA-B,BOLA-DOB,BOLA-DRA*	0.08	Group II
Leukotriene biosynthesis	1.6	*GGT7,LTC4S*	0.14	Group II

^*a*^Terms obtained from Ingenuity Pathway Analysis (IPA).

^*b*^Benjamini–Hochberg adjusted *p*-values <0.05 indicating significance.

^*c*^contribution of the gene dataset (Gene Groups I or II) to respective pathway enrichment, calculated by IPA.

Gene Group II revealed significant enrichment of four pathways that correspond to the immune system, including B Cell Development, IL-4 Signaling and Antigen Presentation Pathway, in overlap to the analysis of ruminal DEGs (Tables 3, 5). Particularly, genes of the Major Histocompatibility Complex (*BOLA-B, BOLA-DOB*, and *BOLA-DRA*) as well as Interferon Regulatory Factor 4 (*IRF4*) contributed to the significant enrichment of immune pathways in Group II (Table 5). *BOLA-DRA* transcripts were significantly less abundant in HMUg compared to LMUg animals (DEG) and revealed positive correlations with abundances of *Roseburia* (*r* = 0.53, *p* = 0.02) and *Lachnospiraceae_FE2018_group* (*r* = 0.52, *p* = 0.02) (Figure 3; Supplementary materials 1, 4). In addition, the presence of Gamma-Glutamyltransferase 7 (*GGT7*) and Leukotriene C4 Synthase (*LTC4S)* in Group II caused the significant enrichment of Leukotriene Biosynthesis Pathway (Table 5). The transcript abundance of both genes was highly correlated with the abundance of *Prevotellaceae_UCG-004* (*GGT7: r* = −0.67, *p* <0.01, *LTC4S*: *r* = −0.52, *p* = 0.02) (Figure 3; Supplementary material 4). Moreover, *GGT7* transcripts showed further a prominent negative correlation to *Lachnospiraceae_UCG-010* abundances (*r* = −0.05, *p* = 0.02), whereas *LTC4S* transcripts negatively correlated with the occurrence of *Coxiella* (*r* = −0.58, *p* <0.01).

Furthermore, *UCG-010_ge* genus attracted interest due to its particularly high correlations with transcript abundances of KH Domain Containing 4 (*KHDC4*; *r* = −0.72, *p* = 0.0003), Ribosomal RNA Processing 9 (*RRP9; r* = 0.71, *p* <0.0005), Lipase Maturation Factor 1 (*LMF1*; *r* = 0.73, *p* <0.0003) and DNA Polymerase Delta 4 (*POLD4*; *r* = 0.73, *p* = 0.0002). Furthermore, *POLD4* expression showed a strikingly high negative correlation with the abundance of *Shuttleworthia* (*r* = −0.74, *p* = 0.00017), whereas *KHDC4* transcripts showed a prominent correlation with *Acetobacter* occurrences (*r* = 0.69, *p* = 0.0008).

## Discussion

In regard to the underlying biological principles of N-metabolism in ruminants, it was hypothesized that the predisposed variance in MU concentration is—in complement to inherent post-ruminal processes—mainly attributed to the ruminal mechanisms that differentiate between HMUg and LMUg cows and thus lead to N-utilization and N-excretion variance in genetically divergent cow cohorts. Specifically, the rumen microbial N-utilization, the absorption and diffusion processes of the rumen epithelium and the ruminal interaction between microbes and host epithelium were thought to characterize the ruminal make up of HMUg and LMUg cows.

### Microbial analyses

Although no differences in the overall microbial diversity have been uncovered between HMUg and LMUg cows, some DAGs were identified. These DAG might (i) influence the amount of ruminal NPN in forms of NH_3_ or ${\text{NH}}_{4}^{+}$, (ii) determine the utilization of blood urea by ureolytic activity, or (iii) influence the permeability for the transport of NH_3_, ${\text{NH}}_{4}^{+}$ and urea across the rumen epithelium.

Specifically, LMUg animals displayed a significantly higher abundance of *Succinivibrionaceae_UCG-002*, which is an ureolytic genus in the rumen. Ureolytic bacteria metabolize urea to CO_2_ and NH_3_. The latter can be further utilized by other ruminal genera for microbial growth, which subsequently reduces the ruminal NPN-pool (Rosendahl, 2015). Moreover, the conversion of urea into NH_3_ increases the urea concentration gradient between blood and the rumen lumen. This massively alters the urea transport rate, reduces blood urea concentration and promotes N-fixation into microbial protein (Rosendahl, 2015; Jin et al., 2016). Interestingly, significantly lower blood urea concentrations and lower blood urea pool sizes were observed in cows displaying the LMU compared to the HMU phenotype in a recent study (Müller et al., 2021). Furthermore, unclassified genera of the *Succinivibrionaceae* family have already been suspected as being more abundant in ruminants with increased N-use efficiency (Jin et al., 2016).

LMUg animals additionally exhibited significantly higher abundances of *unclassified Ruminococcaceae*. Various *Ruminococcaceae* genera correlated with the N-metabolism in goats, including *Ruminococcus_2* displaying significantly higher abundances in animals with higher N utilization efficiency (Wang et al., 2019). Moreover, the *Ruminococcaceae* family has been suggested to play a fundamental role in amino acid and protein metabolism in the bovine rumen, and the *Ruminococcaceae NK4A214 group* has been attributed a role to N-recycling in cattle (Pacífico et al., 2021). Hu et al. (2019) found a positive correlation between the *Ruminococcus* abundances and the gap junctions in the rumen epithelium of yaks and postulated an absorptive response of the rumen epithelium to *Ruminococcus* metabolites. Some *Ruminococcaceae* genera were also reported to have urease activity (Patra and Aschenbach, 2018), and thus it might be conceivable that *unclassified Ruminococcaceae* increases the urea concentration gradient across the rumen wall and support the diffusion of urea in LMUg animals.

Furthermore, HMUg animals showed higher abundances of *Butyvibrio*, which is one of the most relevant butyrate producers in the rumen (Meehan and Beiko, 2014; Henderson et al., 2015; Kong et al., 2020). Interestingly, two *Butyvibrio* species, namely, *B. fibrisolvens* and *B. proteoclasticus*, were postulated as key species involved in protein degradation in the bovine rumen (Wallace and Brammall, 1985; Attwood and Reilly, 1995; Henderson et al., 2015). Due to their high proteolytic activities, they belong to the hyper-ammonia producing bacteria species (HAB) (Hartinger et al., 2018). Also, HAB species are known to negatively influence the N-efficiency of ruminants, since they cause steep increases of NH_3_, which cannot be directly utilized by the rumen microbes and thus leads to enhanced NH_3_ efflux into the bloodstream (Bento et al., 2015; Hartinger et al., 2018). Based on phenotypic categorization into HMU and LMU cow groups, HMU cows were indeed shown to exhibit numerically higher ruminal NH_3_ concentrations than LMU cows (Müller et al., 2021). Furthermore, higher *Butyrivibrio* abundances were found to be correlated with lower N-recycling efficiency in beef cattle (Alves et al., 2020).

The influence of different CP levels on the rumen microbial community has been broadly evidenced (Lapierre et al., 2005; Aguiar et al., 2014; Patra and Aschenbach, 2018; Müller et al., 2021). In general, it is known that lower CP levels in the diets of dairy cows reduce rumen fluid NH_3_ and ${\text{NH}}_{4}^{+}$, enhance the relative blood urea reflux and faster N-assimilation and N-fixation into microbial protein.

In both HMUg and LMUg animals, the reduction of dietary CP content induced significant changes in microbial abundances, which might be caused by microbial N-use competition and the general dietary nutrient availability. In this context, *Sharpea* abundances increased simultaneously with the decline of *Incertae_sedis* abundances in both, HMUg and LMUg cows, when feed-CP content was lowered. *Sharpea* is known to digest short-chain carbohydrates (Dias et al., 2017; Trabi et al., 2020), while *Incertae_sedis* (aggregated genus of the *Ethanoligenenaceae* and *Ruminococcaceae* families) primarily converts cellulose (Suen et al., 2011; Bailoni et al., 2021). The metabolic processes and the resulting microbial growth of cellulose digesters are generally slower than the growth of microorganisms, which metabolize the short-chain sugars. However, both microorganisms need to assimilate N components for their microbial growth (Russell et al., 2009). It might be conceivable that *Sharpea* depressed the growth of *Incertae_sedis* due to comparatively faster N-assimilation, when CP was lowered. *Shuttleworthia* attracted further interest since its abundance increased in HMUg, whereas significant abundance decreases were identified in the LMUg group. Several studies reported feed induced abundance alterations of *Shuttleworthia*, which were related to the dietary starch–fiber ratio and the energy level of the diet (Plaizier et al., 2017; Kotz et al., 2020; Tun et al., 2020). Furthermore, Zhang et al. (2019) postulated significantly different abundances between high and low yielding dairy breeds and identified *Shuttleworthia* as a genus displaying high relative abundances, which is in common with the present study. These findings promote *Shuttleworthia* as highly vulnerable and strongly adaptable genus, which might be of importance in characterizing the different ruminal adaption mechanisms in LMUg and HMUg on low CP levels.

Moreover, the abundances of *Acidaminococcus* and three genera of *Desulfobacterota* phylum (*Desulfovibrio, Desulfobulbus*, and *Desulfobulbaceae_unclassified*) increased in HMUg cows with the reduction of dietary CP content. Interestingly, several species of these genera have been previously categorized as HAB (Attwood and Reilly, 1995; Eschenlauer et al., 2002; Loubinoux et al., 2002). The growth of HAB is known to be suppressed by high NH_3_ and ${\text{NH}}_{4}^{+}$ concentrations in a self-regulating manner (Sales et al., 2000). Interestingly, phenotypic HMU animals displayed significant decreases in previously high ruminal NH_3_ and ${\text{NH}}_{4}^{+}$ concentrations following CP reduction, whereas in LMU phenotypes only moderate ruminal NH_3_ and ${\text{NH}}_{4}^{+}$ declines were observed (Müller et al., 2021). Since genera abundances of the *Desulfobacterota* phylum appeared to be significantly increased exclusively in HMUg, it is conceivable that their growth was inhibited by high ruminal NH_3_ and ${\text{NH}}_{4}^{+}$ levels in HMUg under NP diet feeding, and that the reduction of dietary N might have had a positive effect on their growth.

In LMUg cows, dietary CP reduction led to increased abundances of *Streptococcus*, which is one of the most important proteolytic members in the rumen fluid (Attwood and Reilly, 1995; Ling and Armstead, 1995). *Streptococcus* utilizes rapidly assimilable energy sources (starch), is known to possess ureolytic activity and is highly adaptable to various N-sources, which could provide a competitive advantage under N scarcity (Jin et al., 2016; Hartinger et al., 2018; Patra and Aschenbach, 2018).

Under conditions of LP diet feeding, LMUg cows further exhibited a significant abundance decline of *Acetobacter*, which had already been identified as significantly less abundant in LMUg compared to HMUg cows in this study (contrast HMUg–LMUg). The further massive abundance decline in LMUg cows from NP to LP feeding suggests *Acetobacter* an adaptable capacity to different N levels in the rumen, which may contribute to differential N-utilization in LMUg cows.

### Transcriptome

While the metabolic activity of the microbial community substantially affects the ruminal protein-N to NPN ratio, the gene expression in the rumen villi impact the ruminal milieu (e.g., active and passive transport processes) and thus the composition of the microbial community (e.g., immune modulation) (Penner et al., 2011; Steele et al., 2016). The pathway analysis of DEGs in rumen villi revealed the enrichment of immune response pathways, which were activated in LMUg compared to HMUg cows. Interestingly, it was recently reported in humans that the intestinal mucosa was able to respond to the microbial products with the targeted expression of receptors, immune genes and signaling molecules to maintain intestinal homeostasis (Tannock and Liu, 2020). In addition, the concept of “immune selection” was described as a way of maintaining host–microbe relationships in the long term as a result of host immune activity (Peterson et al., 2007). In this context, Bessman and Sonnenberg (2016) elucidated the essential role of Antigen Presentation and the Major Histocompatibility Complex, which dictated the host T-cell reactivity to biochemical products of commensal bacteria by the expression of innate lymphoid cells. Notably, the highest *Z*-score with significant activation in LMUg compared to HMUg cows was found for the Antigen Presentation Pathway in this study. Furthermore, the pathways of IL-4 Signaling, B Cell Development and Glucocorticoid Receptor identified in LMUg–HMUg contrast were enhanced, predominantly by Bovine Leukocyte Antigens (BOLA), which are prominent drivers of the bovine Major Histocompatibility Complex (Ellis and Ballingall, 1999; Lewin et al., 1999). Moreover, Zhang et al. (2021) reported a simultaneous slight activation of the Antigen Presentation Pathway with a significantly higher expression of *PRRS2* in the rumen tissue of calves with comparatively higher N-efficiency. In fact, *PRSS2* showed a severely higher expression in LMUg compared to HMUg cows. Thus, both antigen presentation and *PRRS2* might constitute key candidates distinguishing ruminal N-utilization of LMUg and HMUg. Also, *PRSS2* has further been associated with feed efficiency and performance traits in cattle (Abo-Ismail et al., 2013), which might be of interest since MX Dynamin Like GTPase 1 (*MX1*) and ISG15 Ubiquitin Like Modifier (*ISG15*) were similarly found to be higher expressed in more feed-efficient heifers, which simultaneously possessed more active immune systems (Paradis et al., 2015). All of these three genes displayed remarkably higher expression levels in LMUg compared to HMUg cows in this study driving the enrichment of Interferon and Neuroinflammation Signaling.

Among the significantly DEG, Matrix Metallopeptidase 3 (*MMP3)* and *TNP2* exhibited the broadest expression differences between cow groups. Metalloproteases were inferred to influence the destruction of tissues by remodeling the extracellular matrix (Coussens et al., 2003; Abendaño et al., 2013) and *MMP3* in specific has been recognized to influence the remodeling of adipose tissue in cattle (Jeong et al., 2017). It might be conceivable that *MMP3* also attributes to structure processes in the rumen tissue and thus contribute to different absorption and diffusion capacities between HMUg and LMUg epithelia, regarding ruminal NH_3_, ${\text{NH}}_{4}^{+}$, and urea fluxes. Furthermore, *TNP2* has been reported in the context of male fertility (Pasquariello, 2015), but up to now, its function in the rumen of cattle as well as its possible role in N-metabolism remains uncertain. However, given the relationship of fertility traits and immune system activities in dairy cows, the differential expression of *TNP2* demands further investigation whether LMUg and HMUg cows differ with respect to fertility parameters (Chebel et al., 2004; Hansen et al., 2004; Moore et al., 2005; König et al., 2008; Weiss et al., 2009; Hurley, 2014).

Although the reduction in dietary CP content did not trigger significantly differential gene expression in rumen tissue of HMUg animals, LMUg cows exhibited severe adaptations in their transcriptome, as characterized by more than 1,000 DEG and 139 significantly enriched pathways. The pathways identified contributed almost exclusively to the immune system, the energy metabolism and the cell cycle. Considering the significantly higher plasma urea pool size during NP compared to LP feeding in cows phenotyped for LMU (Müller et al., 2021), and the general role of immune cells in the rumen wall in protecting the host against infiltration of toxic compounds, the enrichment of these pathways may indicate the necessity for the rumen epithelia to balance the N-gradient and maintain immune homeostasis (Bessman and Sonnenberg, 2016; Tannock and Liu, 2020; Müller et al., 2021). In regard to the severe energy requirements of the immune system (Gleeson et al., 2004; Kvidera et al., 2017), it might further be speculated that the observed effects on energy metabolism pathways in LMUg cows fed the LP ration result from an altered energy demand to stabilize the ruminal N balance.

### Relationship between microbial genera and transcriptome subsets

The correlation analysis between microbial and host gene subsets reflecting the rumen microbiome—host interaction, which distinguished the rumen profiles of HMUg and LMUg cows, identified two main clusters. The pathway analysis of Gene Group I revealed the enrichment of Urea Cycle and Arginine Metabolism, primarily due to increased expression of Arginase 1 (*ARG1*) in LMUg compared to HMUg cows. Arginase is an enzyme that catalyzes the last reaction step in the urea cycle by facilitating the synthesis of arginine into ornithine and urea. In general, the urea cycle is performed predominantly by hepatocytes in the liver (Emmanuel, 1980; Razmi et al., 2005). However, Beck et al. (2009) stated the expression of *ARG1* also in the rumen epithelium of cattle, which implies catabolism of N-metabolites *via* the urea cycle in the rumen wall. Similarly, Emmanuel (1980) identified low *ARG1* expression in the rumen epithelium of sheep. Considering the conversion of NH_3_ into urea, the prominent negative correlation between *ARG1* transcripts and *UCG-005* abundances calls for further research. Although *UCG-005* has not been mentioned directly in the context of N-efficiency in ruminants yet, Amat et al. (2021) predicted *UCG-005* a negative impact on *Methanobrevibacter* abundances in yearling heifers, which is known to contain various N-fixating strains (Poehlein et al., 2018).

Further insights into a possible differentiation of HMUg and LMUg were displayed by Gene Group II, which revealed the enrichment of four immune system pathways, particularly attributed to the expression of *BOLA* genes. Interestingly, *BOLA* polymorphisms were not only reported to determine inherent and acquired immune defenses (Rupp and Boichard, 2003), they were also capable to modulate the composition of colostrum microbiota in dairy cows (Derakhshani et al., 2018). Furthermore, *BOLA* gene expression is known to influence the symbiotic microbial community in the gastrointestinal tract of humans (De Palma et al., 2010), mice (Toivanen et al., 2001), and fish (Bolnick et al., 2014). In the present study, *BOLA-DRA* transcripts were significantly more abundant in LMUg compared to HMUg cows and showed further striking positive correlations to *Roseburia* abundances. The contribution of *Roseburia* to immune responses and to the N-metabolism in cattle, goats, and rabbits has been postulated by various authors (Wang et al., 2011; Alves et al., 2020; Sun et al., 2020). The observed higher abundances of *Roseburia* and *BOLA-DRA* transcripts in LMUg compared to HMUg cows might picture a microbial–host interplay that contributes to the distinction of ruminal patterns between HMUg and LMUg cows by affecting immune response and N-metabolism-associated processes.

In addition to the correlation with *Roseburia, BOLA-DRA* transcripts revealed a severe positive correlation to *Lachnospiraceae_FE2018_group* abundances, which belongs—like as *Roseburia*—to the *Lachnospiracea*e family. *Lachnospiraceae_FE2018_group* had been mentioned in terms of intestinal barrier function, antioxidant balance, and intestinal inflammation in the gut immune system of broilers (Kong et al., 2020; Liu et al., 2021). Moreover, Wang et al. (2021) postulated a significant positive correlation between *Lachnospiraceae family* and intestinal inflammation in dairy cows. The *Lachnospiraceae* family was also found to be significantly more abundant in beef steers with low N-retention efficiency and higher urinary N-excretion (Alves et al., 2020). Furthermore, high abundances of *Lachnospiraceae* were uncovered in the colon and caecum of goats with low N-utilizing phenotype (Wang et al., 2019). Additionally, *Lachnospiraceae* occurrences were found to be correlated with a low feed conversion rate and low feed efficiency in beef steers (Hernandez-Sanabria et al., 2010; Carberry et al., 2012).

## Conclusion

This study focused on the ruminal background of Holstein cows with predisposed higher or lower MU concentration (HMUg–LMUg) as well as on their adaptation to low CP diets. Compared to HMUg predisposition, LMUg cows displayed higher occurrences of ureolytic genera, such as *Succinivibrionaceae_UCG-002* and *Ruminococcaceae*_*unclassified*, which might cause lower blood urea concentrations and lower blood urea pool sizes in LMU phenotypes. Similarly, the higher ruminal NH_3_ concentrations in HMU phenotypes might be attributed to the higher occurrences of high ammonia-producing species hosted by HMUg cows. If enhanced immune responses that were uncovered in LMUg compared to HMUg cows' rumen epithelia influence the epithelial barrier for NPN molecules and thus drive MU phenotype distinction can only be speculated at this point. However, the downregulation of immune responses and energy metabolism pathways in LMUg cows fed a low CP diet might have indicated energy wasting efforts of the rumen epithelia to maintain ruminal N-balance when LMUg cows were exposed to diets with normal CP content. Considering the important role of Arginase in the urea cycle, the observed interplay between *ARG1* expression and *UCG-005* occurrences is specifically proposed for future research on microbe–host interactions. The ruminal patterns identified for HMUg and LMUg cows contribute to a deeper insight into MU predisposition of Holsteins and attribute to the optimization of N-utilization in tandem with the reduction of N-emissions on dairy farms by future breeding selection strategies.

## Data availability statement

The data presented in the study are deposited in the ArrayExpress Microarray Database at EBI (RNA datasets) and in the BioSample Database (16S Amplicon data) repository, accession numbers E-MTAB-9901 (RNA datasets) and PRJNA856508 (16S Amplicon data).

## Ethics statement

The animal study was reviewed and approved by Ethics Committee of the State of Mecklenburg-Western Pomerania (State Office for Agriculture, Food Safety and Fisheries; LALLF M-V7221.3-2-019/19). Written informed consent was obtained from the owners for the participation of their animals in this study.

## Author contributions

CM, MP, and BK organized and carried out animal husbandry. HH and HR jointly collected the samples, performed statistical analysis, and interpreted the data. HH conducted laboratory work and wrote the manuscript. NT performed RNA and 16S sequencing. SP supported data analysis. DS assisted in data interpretation. NR, BK, and KW conceptualized and supervised the study. All authors reviewed the final manuscript and provided critical feedback.

## Funding

As a part of the BlueCow Project (FKZ: 281B101516) this study has received funding from the Federal Ministry of Food and Agriculture.

## Conflict of interest

Author DS was employed by IT-Solutions for Animal Production, Vereinigte Informationssysteme Tierhaltung w.V. (vit). The remaining authors declare that the research was conducted in the absence of any commercial or financial relationships that could be construed as a potential conflict of interest.

## Publisher's note

All claims expressed in this article are solely those of the authors and do not necessarily represent those of their affiliated organizations, or those of the publisher, the editors and the reviewers. Any product that may be evaluated in this article, or claim that may be made by its manufacturer, is not guaranteed or endorsed by the publisher.

## Supplementary material

Supplementary material 1 p. 1*:* Significantly differentially expressed genes (DEG) between HMUg and LMUg cows. Log2fold changes refer to HMUg. p. 2: Gene set for enrichment analysis (*p*-values are below 0.01, and the corresponding *q*-values are below 0.6) between HMUg and LMUg cows. Log2fold changes refer to HMUg.

Supplementary material 2 p. 1: Gene set for enrichment analysis (*p*-values are below 0.01, and the corresponding *q*-values are below 0.6) between NP and LP diet in HMUg cows. Log2fold changes refer to HMUg. p. 2: Gene set for enrichment analysis (*p*-values are below 0.01, and the corresponding *q*-values are below 0.6) between NP and LP diet in LMUg cows. Log2fold changes refer to NP. p. 3: Significantly enriched pathways (adjusted *p*-values are below 0.05) between NP and LP diet in LMUg cows.

Supplementary material 3 p. 1: Microbial subset (53 genera) generated by sparse Partial Least Squares Discriminant Analysis (sPLS-DA) separating HMUg and LMUg cows. p. 2: Gene subset (100 genes) generated by Partial Least Squares Discriminant Analysis (sPLS-DA) separating HMUg and LMUg cows.

Supplementary material 4 p. 1: The *p*-values of the correlation analysis between microbial genera abundances and gene transcripts separating HMUg and LMUg cows (Figure 3). p. 2: Correlation coefficients (*r*-values) of the correlation analysis between microbial genera abundances and gene transcripts separating HMUg and LMUg cows (Figure 3).
